# Supplementary material for: The BiteBarrier transfluthrin emanator demonstrates significant protection against susceptible and resistant malaria and arbovirus vectors in semi-field trials in Tanzania
Source: PLoS One. 2025 Sep 30;20(9):e0320624. doi: 10.1371/journal.pone.0320624 (PMC12483265; doi:10.1371/journal.pone.0320624)
Supplement: S1 Table — (DOCX) [file pone.0320624.s001.docx]

S1 Table: Mosquito recovery rate in the semi-field system for landing and feeding experiments

| Mosquito species | Treatment | Indoor | | Outdoor | |
| --- | --- | --- | --- | --- | --- |
|  |  | Landing (%) | Feeding (%) | Landing (%) | Feeding (%) |
| *An. gambiae* s.s | Control | 72 | 73 | 87 | 87 |
|  | BB | 69 | 64 | 85 | 90 |
| *An. gambiae* s.s (KDR) | Control | 98 | 97 | 100 | 100 |
|  | BB | 78 | 78 | 100 | 100 |
| *An. funestus* | Control | 89 | 92 | 98 | 100 |
|  | BB | 67 | 70 | 88 | 88 |
| *Cx. quinquefasciatus* | Control | 99 | 97 | 100 | 100 |
|  | BB | 90 | 88 | 100 | 100 |
| *Ae. aegypti* | Control | 99 | 97 | 100 | 100 |
|  | BB | 85 | 78 | 100 | 100 |

Recovery rate is defined as the number of each mosquito species recovered of those expected release count. Expected mosquito release count was 2400 for each species indoor and 1200 outdoor for landing and feeding experiments.
