# Supplementary material for: The BiteBarrier transfluthrin emanator demonstrates significant protection against susceptible and resistant malaria and arbovirus vectors in semi-field trials in Tanzania
Source: PLoS One. 2025 Sep 30;20(9):e0320624. doi: 10.1371/journal.pone.0320624 (PMC12483265; doi:10.1371/journal.pone.0320624)
Supplement: S2 Table — (DOCX) [file pone.0320624.s002.docx]

S2 Table: Resistance profile for the different mosquito species tested between December 2023 and February 2024

| Mosquito species | Permethrin (0.75%) | Deltamethrin (0.05%) | $\boldsymbol{\alpha}$-cypermethrin (0.05%) | $\boldsymbol{\lambda}$-cyhalothorin (0.05%) | Bendiocarb  (0.1%) | Pirimiphos methyl (0.25%) |
| --- | --- | --- | --- | --- | --- | --- |
| *An. gambiae* s.s. | 98% | 100% | 100% | 100% | 100% | 100% |
| *An. gambiae* s.s. (KDR) | 88% | 96% | 72% | 66% | 94% | 100% |
| *An. funestus* | 40% | 38% | 13% | 100% | 96% | 100% |
| *Ae. aegypti* | 100% | 100% | 100% | 100% | 96% | 100% |
| *Cx. quinquefasciatus* | 58% | 96% | 30% | 30% | 17% | 1% |
