# Supplementary material for: The BiteBarrier transfluthrin emanator demonstrates significant protection against susceptible and resistant malaria and arbovirus vectors in semi-field trials in Tanzania
Source: PLoS One. 2025 Sep 30;20(9):e0320624. doi: 10.1371/journal.pone.0320624 (PMC12483265; doi:10.1371/journal.pone.0320624)
Supplement: Attachment — Submitted filename: Response to Reviewers.docx [file pone.0320624.s006.docx]

We would like to thank the reviewers for their comments and have taken the following actions to improve the manuscript.

Reviewer #1:

**Comment:** Title: The authors are encouraged to revise the title so that it informs the reader as to what they’ve concluded. The current title simply indicates that they studied the relationship.

**Response**: We appreciate this reviewer’s observation. The title is now revised to: “The BiteBarrier Transfluthrin emanator provides siginficant protection against both susceptible and resistant malaria and arbovirus vectors in semi-field trials in Tanzania”

**Comment:** Abstract, Line 34: The authors (and this reviewer) understand what is meant by ‘outdoor and early biting mosquitoes’, but many readers would not be so well informed. The authors are encouraged to explain what they mean and describe the significance of the temporal and spatial questioning behaviors.

**Response**: We thank the reviewer for this suggestion. The paragraph has been revised accordingly. **Kindly refer to lines 3**9-42.

**Comment:** Line 55: The authors might consider rephrasing the first sentence. Whereas it is true that human population movements and environmental changes may enhance transmission of malaria and arboviruses, new tools and strategies are also needed to reduce risk in endemic areas.

**Response**: We appreciate the reviewer’s suggestion. The sentence is rephrased accordingly. **Please see line 69-70.**

**Comment:** Line 120: The authors stated that treatment and control chambers remained fixed throughout the study to avoid cross-contamination. To what extent might the transfluthrin have become adsorbed or absorbed by fabrics or other components of the chambers? How certain are the investigators that any such residue would not skew the results of subsequent trials/replicates? Had the investigators assessed landing or feeding rates in treated chambers with the BiteBarrier removed? To what extent might the treated panels in one chamber influence activity within an adjacent one?

**Response**. We appreciate the reviewer’s thoughtful comment. The study was conducted in five rounds, each consisting of six replicates, with a three-day washout period following every round. To exclude the potential of carryover effect, on the third day of each washout, mosquitoes were released into all chambers without treatments to assess residual activity. We observed similarly landing rates across all chambers, indicating no residual effect. **Please refer to lines 151-156.**

On whether treatment in one chamber influencing activity in the other chamber, this is unlikely because the chambers are separated by Plywood and polyethene sheet. The control chambers were located adjacent to the treatment chambers, with wind directional movement from the control to the treatment chambers. Kindly see lines **142-143, 145-146.** Also, high feeding rates were observed in the control chambers throughout the study (see S1 Table), suggesting no influence from the treated chambers.

**Comment:** Line 121: The number of volunteers (2 for each group) seems low. Did each volunteer rotate between the different study conditions (indoor vs. outdoor, treatment vs. control)? If not, how can the investigators be certain that certain volunteers were not more – or less – attractive to mosquitoes?

**Response:** Thank you for this important observation. The study included two arms (treatment and control) for each context (indoor or outdoor), requiring a total of four volunteers (two per context). Volunteers rotated between treatment and control conditions within their assigned context to minimize bias. Prior to the study, we conducted preliminary assessments of mosquito attractiveness and found no significant differences among the volunteers. Additionally, the effect of the volunteers was accounted for in the statistical model. **Please see lines 151-156.**

**Comment:** Line 137: The text states that four BB sheets were placed in each of the four corners. This was confusing to this reviewer. The Figure seems to indicate that there was just one BB sheet in each corner.

**Response:** We appreciate the reviewer for pointing this out. The text is revised and now states that “One BB sheet was hung in each corner”. **Please refer to line-176.**

**Comment:** Line 140: How were all mosquitoes collected? Did the collection method cause mortality to any mosquitoes? It would be useful to present the number or proportion of surviving mosquitoes as well as the portion recovered or that had fed.

**Response**: Thank you for the comment. In the feeding experiment, mosquitoes were collected using mouth aspirators (see lines 221-223). This method did not affect survival, as control mortality remained below 5% throughout the study (**line 323**). In the landing experiment, mosquitoes that landed were aspirated, and the remaining mosquitoes were collected using a Prokopack aspirator. These were not held for delayed mortality assessment but were counted to determine total recovery.

The number of fed and total recovered mosquitoes is presented in Table 2, and the proportion recovered is provided in S1 Table. Additionally, the volunteers participating in these experiments possess extensive experience in handling mosquitoes. Their background includes training in various techniques for mosquito capture, identification, and management, which significantly enhances the reliability and efficiency of our study.

**Comment:** Line 147: The authors stated that mosquito density was increased to 80 per strain for each replicate. But they stated earlier (Line 141) that 80 mosquitoes of each strain were used. This is confusing.

**Response**: Thank you for the comment. We confirm that both Line 141 (now 180) and Line 147 (now 182) consistently state that 80 mosquitoes per strain were used in each replicate.

**Comment:** Line 154: Did the investigators measure wind speed and direction? If so, how? Where were the volunteers situated relative to the BBs and prevailing air flow?

**Response**: We appreciate the comment. Wind speed was measured at 0.0 m/s (lines 115-116) in the partitioned semi-field chambers and was therefore considered negligible.

**Comment:** Line 156: Why were only 40 mosquitoes released for the outdoor tests?

**Response:** Thank you for the question. The decision to release 40 mosquitoes in the outdoor tests was based on power calculations to detect differences in landing rates between treatment and control arms. For indoor tests, we released 80 mosquitoes to maintain statistical power, as preliminary experiments showed that only about 50% of mosquitoes typically enter the hut within one hour in the absence of treatment (lines 186-188).

**Comment:** Line 206: What amount of blood within a mosquito would qualify it to be considered as fed? How was the extent of bloodfeeding assessed?

**Response**: We appreciate the question. Any visible blood in the abdomen was considered evidence of feeding. Blood-feeding was assessed manually by visually inspecting each mosquito individually under adequate lighting in the laboratory.

**Comment:** Line 215: The description of protection efficacy is confusing to this reviewer, and likely would be beyond comprehension by readers not familiar with landing and feeding assays.

**Response**: Thank you for the comment. Protective efficacy was calculated as (1 − odds ratio) × 100, where the odds ratios (ORs) were derived from mixed-effects logistic regression models. This is clearly stated in **lines 239-247**. The models adjusted for potential confounders, including method (landing vs. feeding), treatment, context (indoor vs. outdoor), volunteer, chamber, experimental night/replicate, temperature, and humidity. In line 252 and table 2 we have clarified that this is a reduction in the odds of feeding.

**Comment:** Table 1: The actual numbers of mosquitoes landing or feeding should ideally be displayed.

**Response:** We appreciate this concern. The number of landing and feeding as well as total recovered mosquitoes in the treatment chambers are displayed in Table 1.

**Comment:** Table 2: The authors have seemingly provided an aggregate of the number of recovered and blood-fed mosquitoes across all replicates. How much variation occurred between replicates? Were some volunteers more attractive than others to mosquitoes?

**Response**: Thank you for the comment. We aggregated data in Table 2 to present a clear summary, as displaying results for all 30 replicates per strain would be challenging. Minimal variations between some replicates existed and this was accounted for in the statistical model. Differences in volunteer attractiveness were not significant and were also accounted for in the statistical models (lines 317-319, Table 2 footnote).

**Comment:** Line 254: This sentence seems to imply that there was greater mortality among pyrethroid-resistant mosquitoes. Table 3 suggests otherwise.

**Response**: We appreciate this observation. The sentence has been changed and now reads “Mortality was higher among malaria vectors than arbovirus vectors (Table 3) and appeared to be related to susceptibility levels”. Please see line 325.

**Comment:** Line 285: Mosquitoes can transmit pathogens, but they cannot transmit diseases. The authors are encouraged to avoid colloquial phrasing regarding ‘disease transmission’ by mosquitoes. Infectious mosquitoes that are probing and salivating may transmit arboviruses as well as sporozoites of Plasmodium. Accordingly, a landing rate may be of greatest significance to assessments of transmission of pathogens from mosquitoes to people. In contrast, feeding rates are more relevant to assessments of uptake of pathogens by mosquitoes. The authors are encouraged to elaborate on the discussion of their data as they relate to these features of transmission dynamics.

**Response**: Thank you for this insightful comment. We agree with the reviewer’s clarification that mosquitoes transmit pathogens, not diseases and we have revised the text accordingly. We have also expanded the discussion to better reflect the biological significance of both landing and feeding metrics. Specifically, we now clarify that landing rate is a relevant proxy for pathogen transmission to humans during probing, while feeding rate is more directly related to pathogen uptake by mosquitoes (see lines 366-373).

**Comment:** Line 292: The authors state that recovery rates were ‘below or exceeded the estimated release mosquito count’. This statement is troubling to this reviewer, as the authors mentioned earlier that they released 40 or 80 mosquitoes per replicate. It should have been easy and straightforward to precisely count the 40 or 80 mosquitoes for release. It is understandable that a few released mosquitoes might have been elusive and not recovered at the end of each replicate. When the recovery rates exceeded the estimated release count, could the authors comment on whether the recovered mosquitoes among living or dead? Did they use different colored fluorescent tags that might allow them to assess the species or group to which they belonged? Perhaps, the authors might have increased the interval between the replicates to better ensure that live mosquitoes released during an earlier trial would not confound results of a subsequent replicate.

**Response**: Thank you for raising this important point. While the target number of mosquitoes per replicate was 40 or 80, minor deviations are occasionally expected during packing, especially when handling multiple species. We have clarified this further by adding a phrase reading; “Instances where the number of recovered mosquitoes exceeded the release count were rare and did not significantly affect overall recovery patterns across replicates. We acknowledge that, despite a one-hour collection period, some mosquitoes may temporarily remain hidden in the large semi-field system despite that we lowered the roof and covered the floor with white tarpaulin to enhance visibility” (line 380-384). Also, the mosquitoes that could have remained in the cage were collected in the morning using prockopack aspirators.

The term recovered as defined in line 263 means all mosquitoes whether dead or alive that were captured by all collection methods after the experiment.

Regarding coloring, among the two *An. gambiae* strains morphologically similar, one was marked with fluorescent powder to distinguish resistant from susceptible strains (line 126-130). The other species were morphologically distinct and did not require marking.

**Comment:** Line 336: The authors acknowledge the need for further investigations beyond eight weeks of product aging. Why was the eight-week interval selected for this study? What is the expected duration useful life of this product?

**Response**: We appreciate the question. The eight-week aging period was based on preliminary guidance from the manufacturer regarding the expected effective duration of the BiteBarrier. Given that we observed substantial protective efficacy even after eight weeks, we recommend further longitudinal evaluations to better determine the full duration of efficacy under different environmental conditions.

**Comment:** Figures: These do not impart sufficient information to justify their inclusion.

**Response**: The figure has been revised to include the outlook of the semi-field system.

Reviewer #2:

**Comment:** This study evaluates the protective efficacy of the BiteBarrier transfluthrin emanator against malaria-transmitting vectors. The choice of mosquito species and the study duration represent important contributions to the field. The use of blood-feeding reduction as an indicator of protection—and its comparison to landing-based assessments—raises relevant questions about well established methods such as HLC.
To enhance the reader’s understanding of the environmental context, we recommend including photographs of the study sites. The current Figure 1 and its accompanying description do not adequately depict key aspects of the setting, such as surrounding vegetation and wind barriers. These features are critical, as wind conditions—discussed in the manuscript—can substantially influence both the performance of spatial repellents and mosquito behavior.

**Response**: We thank the reviewer for the positive acknowledgment of our study’s contributions and the insightful comments regarding the environmental context and visual representation of the study site. We have revised the figure to include a photograph of the semi-field system and its surrounding vegetation. Additionally, the environmental conditions during the study period are provided (see line 114-116).

**Comment:** Additionally, we suggest that the authors expand the definition of protective efficacy, specifying how it was calculated and applied within the study. In particular, it would be helpful to clarify how the relative protective efficacy was derived from the comparison of mosquito landing and feeding outcomes. This clarification is essential for a full understanding of the results presented in Table 1.

**Response**: Thank you for the comment. Protective efficacy was calculated as (1 − odds ratio) × 100, where the odds ratios (ORs) were derived from mixed-effects logistic regression models. This is stated in line 239-247. The models adjusted for potential confounders, including method (landing vs. feeding), treatment, context (indoor vs. outdoor), volunteer, chamber, experimental night/replicate, temperature, and humidity.
